# Microbial community composition of Tirez lagoon (Spain), a highly sulfated athalassohaline environment

**DOI:** 10.1186/2046-9063-9-19

**Published:** 2013-10-02

**Authors:** Lilia Montoya, Carlotta Vizioli, Nuria Rodríguez, María José Rastoll, Ricardo Amils, Irma Marin

**Affiliations:** 1 IPICYT, División de Biología Molecular, Instituto Potosino de Investigación Científica y Tecnológica, San Luis Potosí, SLP 78216, México; 2 Departamento de Biología Molecular, Edificio de Biología, Universidad Autónoma de Madrid, Cantoblanco, 28049, Madrid, Spain; 3 Centro de Astrobiología (INTA-CSIC), Torrejón de Ardoz, 28850, Madrid, Spain; 4 Centro de Biología Molecular Severo Ochoa (UAM-CSIC), Cantoblanco, 28049, Madrid, Spain

## Abstract

**Background:**

The aim was to study the seasonal microbial diversity variations of an athalassohaline environment with a high concentration of sulfates in Tirez lagoon (La Mancha, Spain). Despite the interest in these types of environments there is scarce information about their microbial ecology, especially on their anoxic sediments.

**Results:**

We report the seasonal microbial diversity of the water column and the sediments of a highly sulfated lagoon using both molecular and conventional microbiological methods. Algae and *Cyanobacteria* were the main photosynthetic primary producers detected in the ecosystem in the rainy season. Also dinoflagelates and filamentous fungi were identified in the brines. The highest phylotype abundance in water and sediments corresponded to members of the bacterial phylum *Proteobacteria*, mainly of the *Gamma-* and *Alphaproteobacteria* classes. *Firmicutes* and *Actinobacteria* were isolated and identified in Tirez brines and sediment samples. Halophilic sulfate reducing *Deltaproteobacteria* were also detected (*Desulfohalobium*).

**Conclusions:**

Important differences have been found in the microbial diversity present in the Tirez water column and the sediments between the wet and dry seasons. Also the Tirez lagoon showed a high richness of the bacterial *Alpha-* and *Deltaproteobacteria*, *Bacteroidetes*, *Firmicutes*, *Actinobacteria* and for the archaeal *Euryarchaeota*.

## Background

Identifying the limits of life is a major question in microbial ecology. The current exploration of life on Earth has led to the discovery of living systems in environments that were considered inhabitable only few years ago. Thus over the past several years, knowledge of the microbial diversity and ecology of extreme environments has become a vital tool both to answer fundamental questions regarding life’s adaptation to extreme conditions and also to explore the biotechnological potential of extremophiles.

**Figure 1:**
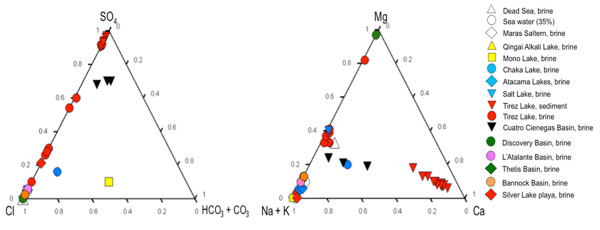
**Chemical composition compared from diverse hypersaline lakes following the Eugster and Hardy criteria**[8]**.** Dead Sea, brine [10], Sea water [2], Maras Saltern, brine [11], Qingai Alkali Lake, brine [12], Mono Lake, brine [13], Chaka Lake, brine [14], Atacama Lake, brine [15], Salt Lake, brine [10], Tirez Lagoon, sediment and brine from present study (multiple samples represent different depths or different seasons), Cuatro Cienengas Basin, brine [16], Discovery Basin, brine [17], L’Atalante, Thetis and Bannock basins [18] and Silver Lake playa lake [19].

Hypersaline environments can be classified as thalassohaline (marine composition) and athalassohaline (non marine composition). The best-characterized are the thalassohaline environments. There is a broad diversity of athalassohaline systems, *e.g.* haloalkaline, with predominance of bicarbonate and sodium ions [1]; some hypersaline anoxic basins and ancient evaporite deposits with salts mainly composed of MgCl_2_[2]. Although several studies have been conducted to understand activities such as sulfate reduction under hypersaline conditions [3-6], there has been less effort to characterize the associated microbial diversity [2,4,7]. Figure 1 shows different types of hypersaline systems based on the classification of Eugster and Hardie [8]. The extreme high concentration of sulfates observed in Tirez lagoon makes it an interesting location to study the effect of sulfate concentration on the microbial diversity of the habitat, especially in the sediments, a halophilic environment that has been poorly explored [9].

**Figure 2:**
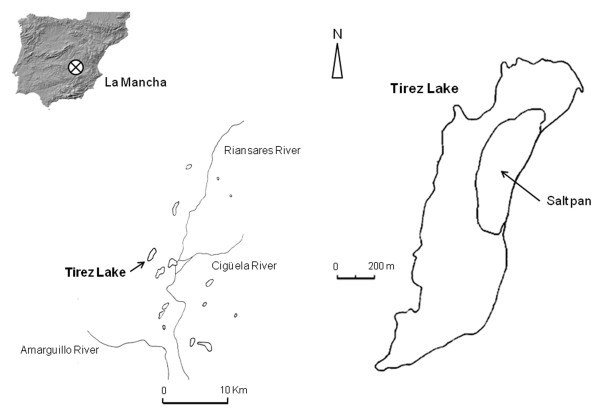
Map showing the Tirez lagoon sampling site (UTM: 4377309, 46982) in La Mancha region (Spain).

Tirez lagoon is located in the southern subplateau of central Spain’s La Mancha region (Figure 2) and is one of several endorheic hypersaline lagoons originated under semiarid conditions in the Iberian Peninsula. These habitats are environmentally important wetlands, which led UNESCO to grant them the status of “Biosphere Reserve” in 1981. Tirez comprises an area of 0.8 km^2^ and its salt composition is determined by inflow water coming from Triassic evaporites and dolomites and calcium-sulfate marls from Tertiary age [20,21]. Salt content in the water column can reach values as low as 6.9% (w/v), during the rainy season, to saturation, in early summer, when high concentrations of SO_4_^2-^, Cl^-^, Ca^2+^ and Mg^2+^ are found [20]. Contrasting with low concentrations of CO_3_^2-^, which probably comes from biological processes, which produce CO_2_[22]. This condition promotes the crystallization of salts such as halite (NaCl), epsomite (MgSO_4_·7H_2_O), mirabilite (Na_2_SO_4_·10H_2_O), polyhalite (K_2_MgCa_2_(SO_4_)·2H_2_O), thenardite (Na_2_SO_4_), hexahydrite (MgSO_4_·7H_2_O) and bloedite (Na_2_Mg(SO_4_)_2_·4H_2_O) [20]. In these conditions, few or no carbonates are present in the sediment, leading to a neutral pH. The dynamics of this saline lagoon is heavily influenced by the semiarid climate. The annual mean temperature is 14.8°C, the mean annual rainfall is ~400 mm, mostly during spring and fall. In the summer, the dry conditions and high temperatures generate the complete evaporation of the lagoon. Tirez has been proposed as a terrestrial analogue of Europa’s ocean, based on the hydrogeochemical characteristics of the lagoon and its similarity with the Galileo’s Near Infrared Mapping Spectrometer data from Europa’s surface [20].

The purpose of the present study was to generate a broad overview of the biodiversity present in the different phases of Tirez lagoon, a sulfated athalassohaline environment, and their seasonal variation. Therefore, the communities dominating the sediment and water phases in the wet and dry seasons were analyzed by performing a combination of microscopy observation, culture-dependent and -independent techniques.

## Results

### Physico-chemical characterization

The physicochemical characteristics of Tirez samples, salinity in particular, were significantly different between the rainy and the dry seasons (Table 1). Salinity in the water column ranged from 6.9%(w/v) to saturation. The redox potential values measured in the water column ranged from +151 mV at low ionic strength to +174 mV at high ionic strength. The corresponding values measured in the sediment cores varied between -282 mV in the wet season and -225 mV in the dry season. The pH of the water in the dry season had a mean value of 7.3 while in the rainy season the water was alkaline (9.08). The sediments pH values were close to neutral with 6.9 being the lowest value during the dry season and 7.8, the highest, in the rainy season. The maximum water depth measured in the rainy season was 40 cm and the mean depth was around 25 cm. Temperature in the brines was 10.1°C in winter and 23.8°C in the summer. The oxygen concentration in the sediments was 0.7 μM in the winter and 0.9 μM in the summer.

**Table 1:** Physico-chemical parameters of water column and sediments in Tirez lagoon

	**Winter**	**Summer**
Salinity (%) (water)	6.9	Saturation
Eh (mV) (water)	+151	+174
Eh (mV) (sediment)	-282	-225
Temperature (°C) (water)	10.1	23.8
pH (water)	7.3	9.08
pH (sediment)	7.8	6.9
Oxygen (μM) (sediment)	0.7	0.9
Prefix of isolates, bands and clones (water)	Rw	Dw
Prefix of isolates, bands and clones (sediment)	Rs	Ds

### Enumeration of cells in water and sediment

The total DAPI cell count in the water column from the rainy season was 2.6×10^8^ ± 0.8x10^8^ cells/ml. At high ionic strength cell numbers dropped one order of magnitude. In the sediments the maximum cell counts were detected at a depth of 10 cm and corresponded to 4×10^7^ ± 0.4×10^7^ cells/g of wet sediment. At deeper zones cell numbers decreased drastically reaching values of 8×10^3^ ± 0.8x10^3^ cells/g of wet sediment.

### Microscopy observation of water samples

Observation of the low ionic strength samples fixed with formaldehyde allowed the morphotypes of the main photosynthetic primary producers present in the system to be identified: members of *Dunaliella* (*Chlorophyta*) and cyanobacteria genera (*Nodularia*, *Microcoleus*, *Anabaena*, *Nostoc* and *Pseudoanabaena*) (Table 2, Additional file 1: Figure S1). In some of the low ionic strength samples the rotifer *Hexartra* sp. and the arthropod *Artemia* sp., common in other saline systems [23,24], were also observed (Table 2). At high ionic strength only *Dunaliella* sp. was detected.

**Table 2:** Microorganisms present in Tirez lagoon water column and sediments identified by microscopic observation, clone-sequencing, DGGE band-sequencing and sequencing of culture isolates

**ID (Access no.) **^**a**^	**Closest relative **^**b**^	**No. of occurrences **^**c**^			
		**Rw**	**Dw**	**Rs**	**Ds**
Bacteria					
*Actinobacteria*					
Rw_cb_7 (FJ172065)	Uncultured actinobacterium clone TDNP_LSbc97_3_28_94 (FJ516859). Semiarid wetland (Central Spain) [97%]	1			
	*Microbacteriaceae* [100%], *Cryobacterium* [57%]				
Rw_db_11 (EU722702)	Uncultured organism clone SBYG_2070 (JN450257). Guerrero Negro hypersaline microbial mat [99%]	1			
	Actinomycetales [100%], *Microbacteriaceae* [97%], *Schumannella* [28%]				
Rs_ib_2A (EU722709)	*Arthrobacter phenanthrenivorans* strain Sphe3 (NR_074770) [97%]			2	
	*Micrococcaceae* [100%], *Arthrobacter* [49%]				
Bacteroidetes					
Rw_cb_8 (EU725593)	Uncultured marine bacterium clone BM1-F-27 (FJ826125). Yellow Sea [97%]	1			
	Flavobacteriales [100%], *Flavobacteriaceae* [97%]				
Ds_db_31 (EU722701)	Uncultured bacterium clone BJGMM-1s-364 (JQ800813). Soil samples from the Yellow River DeltaYellow River [98%]				1
	*Flavobacteriaceae* [100%], *Gillisia* [99%]				
Rs_cb_74 (EU722650)	Uncultured bacterium clone a43 (HM468007). Wastewater treated with with ferrous salt [99%]			1	
	Sphingobacteriales [100%], *Cyclobacteriaceae* [81%], *Nitritalea* [81%]				
Ds_db_28 (EU722700)	*Muricauda flavescens* strain SW-62 (NR_042908). Salt lake, Korea [82%]				1
	Bacteria [100%], Bacteroidetes [87%]				
Rs_cb_66 (EU722655)	Uncultured organism clone MAT-CR-P2-E05 (EU246052). Hypersaline microbial mat [96%]			1	
	*Marinilabiaceae* [100%], *Anaerophaga* [99%]				
Rs_db_2 (FJ172074)	Uncultured bacterium clone 184-32 (GU212609). Saline soil samples Qaidam Basim, China [97%]			1	
	*Marinilabiaceae* [100%], *Anaerophaga* [66%]				
Flavobacteria					
Rs_db_12 (EU722690)	Uncultured Bacteroidetes bacterium clone CF07-19 (FJ844036). High mountain lake, China [93%]				1
	*Flavobacteriaceae* [100%], *Psychroflexus* [55%]				
Rw_cb_19 (EU725598)	*Psychroflexus sediminis* strain YIM C238 (NR_044410). Haloalkaline soil [98%]	1			
	*Flavobacteriaceae* [100%], *Psychroflexus* [100%]				
Rs_cb_27 (EU722652)	*Psychroflexus sediminis* strain YIM C238 (NR_044410). Haloalkaline soil [98%]			2	
	*Flavobacteriaceae* [100%], *Psychroflexus* [100%]				
Rs_db_7 (EU722686)	Uncultured *Salegentibacter* sp. clone HAHS13.025 (HQ397000). Haloalkaline soil [98%]	2		5	
	*Flavobacteriaceae* [100%], *Salinimicrobium* [95%]				
Rw_cb_17 (EU725597)	*Psychroflexus sediminis* strain YIM C238 (NR_044410). Haloalkaline soil [98%]	1			
	*Flavobacteriaceae* [100%], Psychroflexus [100%]				
Rs_cb_80 (EU722656)	Uncultured bacterium clone MBFOS-06 (EU369165). Oyster shell [95%]			1	
	*Flavobacteriaceae* [100%], *Salinimicrobium* [99%]				
Sphingobacteria					
Ds_db_20 (EU722696)	Sphingobacteria bacterium clone A1503 (EU283512). Anderson lake [95%]				1
	Bacteroidetes [100%], Flavobacteria [50%]				
Bacilli					
Rs_db_13 (EU722691)	Uncultured bacterium clone H0014 (JX391054). Marine sediment [90%]			2	1
	Firmicutes [99%], Bacilli [93%], Bacillales [81%]				
Rs_db_4 (FJ172072)	Uncultured bacterium clone B-2 (HQ703872). Qinghai lake sediment [98%]			1	
	Bacteria [100%], Firmicutes [92%], Bacilli [65%]				
Ds_db_22 (EU722698)	Uncultured bacterium clone Kasin-B2-B05 (HE604654). Hypersaline sediments [99%]			1	
	*Sporolactobacillaceae* [100%], *Sporolactobacillaceae* incertae sedis [100%]				
Ds_ib_4 (EU722711)	*Bacillus* sp. DHC09 (JQ904720). Sea surface sediment [97%]				1
	Bacillales [100%], *Bacillaceae* 1 [80%], *Falsibacillus* [71%]				
Rs_ib_3A (FJ172083)	*Paenibacillus* sp. 5-3 (HQ832503) Food waste [92%]			2	
	Bacilli [97%], Bacillales [97%], *Paenibacillaceae* 1 [80%]				
Rs_ib_4iA (FJ172086)	*Paenibacillus* sp. ITCr59 (FR823415). Agricultural soil [97%]			5	
	*Paenibacillaceae* 1 [100%], *Fontibacillus* [95%]				
Clostridia					
Rs_db_29 (FJ172073)	Uncultured bacterium clone H3034 (JX391174). Marine sediment [98%]				1
	Clostridiales [100%], *Clostridiaceae* 3 [99%], *Sporosalibacterium* [96%]				
Alphaproteobacteria					
Rw_cb_4 (EU725591)	Uncultured Rhodobacteraceae clone DS127 (DQ234210). River estuary [98%]	1			
	*Rhodobacteraceae* [100%], *Roseovarius* [86%]				
Rw_cb_6 (FJ172064)	*Roseobacter* sp. B11 (DQ659411) [99%]	2			
	*Rhodobacteraceae* [100%], *Seohaeicola* [98%]				
Rw_cb_2 (FJ172062)	*Loktanella vestfoldensis* strain R-9477 (NR_029021) [98%]	12			
	*Rhodobacteraceae* [100%], *Loktanella* [100%]				
Gammaproteobacteria					
Rs_cb_73 (EU722649)	Uncultured *Idiomarina* sp. clone XJ120 (EF648161). Aerobic activated sludge [94%]			1	
	*Idiomarinaceae* [100%], *Pseudidiomarina* [99%]				
Rs_cb_1 (FJ236711)	*Pseudidiomarina* sp. YCSA4 (GQ246209). Water of a salt field [93%]			3	
	*Idiomarinaceae* [100%], *Pseudidiomarina* [100%]				
Ds_ib_6 (FJ172080)	*Pseudidiomarina homiensis* strain CT34 (HM854277) [99%]				4
	Idiomarinaceae [100%], *Idiomarina* [81%]				
Rs_cb_93 (EU722651)	Uncultured proteobacterium clone TY4SP11r (JQ218797). Marine macro-alga [95%]			1	
	*Idiomarinaceae* [100%], *Pseudidiomarin*a [98%]				
Rs_cb_26 (EU722643)	*Pseudidiomarina* sp. 2PR54-15 (EU440967) [96%]			9	
	*Idiomarinaceae* [100%], *Idiomarina* [92%]				
Ds_ib_11 (EU722714)	Uncultured gamma proteobacterium clone XJ85 (EF648142). Aerobic activated sludge [98%]				1
	*Idiomarinaceae* [100%], *Pseudidiomarina* [59%]				
Ds_ib_3 (EU722710)	*Marinobacter adhaerens* strain S20-1 (KC420687) [100%]			1	1
	*Alteromonadaceae* [100%], *Marinobacter* [100%]				
Rs_db_16 (EU722694)	Uncultured bacterium clone SN18 (JQ824910). Saline and alkaline soil [97%]			1	
	Gammaproteobacteria [99%], Alteromonadales [52%], *Alteromonadaceae* [52%]				
Rw_cb_9 (FJ172066)	Uncultured bacterium clone SN26 (JQ824918). Saline and alkaline soil [98%]	1		1	
	*Piscirickettsiaceae* [100%], *Methylophaga* [100%]				
Rw_cb_46 (FJ172063)	Uncultured bacterium clone SINP962 (HM127832). Qinghai lake [91%]	1			
	Bacteria [100%], Proteobacteria [61%], Gammaproteobacteria [61%]				
Rs_cb_94 (FJ236710)	Uncultured bacterium clone Lupin-1130m-2-pse1 (EF200114). Subpermafrost fracture waters in Artic [94%]			1	
	Proteobacteria [99%], Gammaproteobacteria [98%], Oceanospirillales [47%]				
Dw_ib_7 (EU722712)	*Pseudoalteromonas* sp. TA010_3 (EU308473). Solar saltern [98%]		1		
	*Pseudoalteromonadaceae* [100%], *Pseudoalteromonas* [100%]				
Rs_db_10 (EU722688)	*Halomonas* sp. HL33 (KC705271). Hot lake hypersaline margin soil [95%]			3	1
	*Halomonadaceae* [100%], *Cobetia* [41%]				
Ds_ib_8 (EU722713)	Uncultured *Halomonas* sp. clone BPS_CK65 (HQ857613). Hydrocarbon contaminated saline [98%]				9
	*Halomonadaceae* [100%], *Halomonas* [98%]				
Rs_cb_64 (FJ172071)	*Halomonas sediminis* strain YIM C248 (EU135707) [98%]			1	
	*Halomonadaceae* [100%], *Halomonas* [83%]				
Rs_db_15 (EU722693)	Uncultured bacterium clone 100307_0m_01F (KC358335). Low salinity soda lake [95%]			1	
	*Piscirickettsiaceae* [100%], *Thioalkalimicrobium* [94%]				
Deltaproteobacteria					
Rs_db_srb (EU722708)	Uncultured bacterium clone E6bG07 (DQ103666). Hypersaline endoevaporitic microbial mat [96%]			1	
	*Desulfohalobiaceae* [100%], *Desulfohalobium* [82%]				
Cyanobacteria					
	*Nostoc* sp. (*)	*			
	*Anabaena* sp. (*)	*			
	*Pseudoanabaena* sp. (*)	*			
	*Nodularia* sp. (*)	*			
Rw_ib_C (FJ172091)	*Leptolyngbya* sp. LEGE 07084 (HM217072). Temperate estuary [98%]	2			
	Cyanobacteria [100%], Family IV [100%], GpIV [100%]				
Rw_db_6 (FJ172075)	Uncultured organism clone SBXY_5108 (JN429822). Hypersaline microbial mat [99%]	1			
	Cyanobacteria [100%], Family XIII [78%], GpXIII [78%]				
Archaea					
Halobacteria					
Rs_ca_8 (EU722666)	Uncultured haloarchaeon clone XKL10 (JN714413). Saline lake [99%]			1	
	*Halobacteriaceae* [100%], *Halobacterium* [46%]				
Rs_ca_62 (EU722680)	Uncultured haloarchaeon clone XKL44 (JN714440). Saline lake [98%]			3	
	*Halobacteriaceae* [100%], *Halolamina* [100%]				
Rs_ca_16 (EU722670)	Uncultured haloarchaeon clone XKL11 (JN714414). Saline lake [96%]			1	
	*Halobacteriaceae* [100%], *Halococcus* [99%]				
Rs_ca_29 (EU722669)	Uncultured haloarchaeon clone XKL23 (JN714423). Saline lake [96%]			1	
	*Halobacteriaceae* [100%], *Halomicrobium* [88%]				
Rs_ca_64 (EU722681)	Halobacteriaceae archaeon EA3 (HQ197981). Salt lake brine [99%]			1	
	*Halobacteriaceae* [100%], *Halolamina* [100%]				
Rs_ca_10 (EU722677)	Halobacteriaceae archaeon R30 (HM159607). Salted kelp [97%]			1	
	*Halobacteriaceae* [100%], *Halonotius* [97%]				
Rs_ca_7 (EU722667)	Uncultured *Halobacterium* sp. clone 7A23 (AY987826). Maras salterns [99%]			2	
	*Halobacteriaceae* [100%], *Halobacterium* [100%]				
Dw_ca_51 (FJ172059)	*Halobacterium* sp. AUS-2 (D32082) [97%]		2		
	*Halobacteriaceae* [100%], *Halorubrum* [100%]				
Rs_ca_5 (EU722663)	Uncultured euryarchaeote clone DSFBPENV12arc_7C (KC465576). Brine pool water [99%]			7	
	*Halobacteriaceae* [100%], *Halobacterium* [66%]				
Ds_da_7 (FJ172052)	Uncultured archaeon clone Kasin-A3-B06 (HE604580). Exposed salt lake sediment [99%]				1
	Halobacteriaceae [100%], *Salarchaeum* [56%]				
Rs_ca_49 (EU722671)	Uncultured haloarchaeon clone TX4CA_35 (EF690590). Alkaline-saline soil [94%]			1	
	*Halobacteriaceae* [100%], *Halococcus* [69%]				
Dw_ca_34 (FJ172057)	*Halorubrum kocurii* (AB576124) [99%]		4	2	
	*Halobacteriaceae* [100%], *Halorubrum* [100%]				
Dw_ca_36 (FJ172058)	*Halorubrum xinjiangense* strain BD-1 (NR_028205) [99%]		1		
	*Halobacteriaceae* [100%], *Halorubrum* [100%]				
Dw_ca_59 (FJ172060)	Uncultured euryarchaeote clone SFH1E051 (FN391283). Solar saltern sediment [99%]		1		
	*Halobacteriaceae* [100%], *Halorhabdus* [43%]				
Rs_ca_31 (EU722657)	Uncultured haloarchaeon clone XKL48 (JN714443). Saline lake [99%]			1	
	*Halobacteriaceae* [100%], *Natronomonas* [100%]				
Rs_ca_59 (EU722658)	Uncultured haloarchaeon clone XKL48 (JN714443). Saline lake [99%]			1	
	Halobacteriaceae [100%], *Natronomonas* [100%]				
Ds_da_3 (EU722706)	*Natronobacterium* sp. isolate 2-24-8 (AJ878084) [97%]				1
	*Halobacteriaceae* [100%], *Natronomonas* [100%]				
Rs_ca_93 (EU722674)	*Halomicrobium katesii* (JN120802) [99%]			1	
	*Halobacteriaceae* [100%], *Halomicrobium* [100%]				
Rs_ca_52 (EU722672)	*Haloarcula* sp. AB19 (DQ471854) [99%]			1	
	*Halobacteriaceae* [100%], *Haloarcula* [100%]				
Rs_ca_21 (EU722673)	Halophilic archaeon strain BNERC31 (AB766180). Solar saltern [96%]			1	
	*Halobacteriaceae* [100%], *Haloarcula* [100%]				
Archaeoglobi					
Rs_da_2 (EU722705)	Uncultured archaeon clone 11 (GQ452803). Hypersaline methane seep in canadian high Artic [97%]			2	
	Archaeoglobales [98%], *Archaeoglobaceae* [98%], *Archaeoglobus* [63%]				
Candidate division MSBL1					
Rs_ca_41 (EU722682)	Uncultured euryarchaeote clone Discovery_a (HQ530525). Hydrothermal brine system in the Red Sea [93%]			1	
	Euryarchaeota [96%], Archaeoglobi [49%], Archaeoglobales [49%]				
Eukarya					
Bascilariophyceae					
Rw_ie_diat (FJ172078)	*Nitzschia communis* strain FDCC L408 (AJ867278) [99%]	1			
Monogononta					
*Hexarthra* sp. (*)		*			
Branchiopoda					
*Artemia* sp. (*)		*			
Chlorophyceae					
*Dunaliella* sp. (*)		*	*		
*Dinophyceae*					
Rw_ie_din (EU734574)	*Woloszynskia cincta* strain MALINA FT56.6 PG8 (JN934667) [98%]	1			
Eurotiomycetes					
*Aspergillus* sp. (*)					*

a) The suffixes define technique of identification and Domain: clone library of Bacteria (cb), DGGE gel band of Bacteria (db), isolate of Bacteria (ib), clone library of Archaea (ca), DGGE gel band of Archaea (da) and microscopic identification (*).

b) The sequence identity (%) was determined by two methods: BLASTN in the GenBank database (first line of the cell) [25] and *Classifier* in the RDP (second line of the cell) [26].

c) The prefixes define the origin of the samples: (Dw) Water from dry season, (Rw) Water from rainy season, (Rs) Sediment from rainy season, (Ds) Sediment from dry season.

### Isolation from enrichment cultures

#### Water isolates

A 5 mm thick granular green biofilm with a grey texture covering the sediments in the rainy season was inoculated in specific cyanobacterial growth media. One isolate, Rw_ie_diat (*Bacillariophyta*) had 99% 16S rRNA gene sequence similarity with the chloroplast from the diatom *Nitszchia communis* (Table 2). *Nitszchia* species are frequently found in hypersaline systems [23] whereas two other isolates, Rw_ib_C and Rw_ib_D, showed 97% similarity with *Leptolyngbya* sp. (Table 2). Only one heterotrophic bacterium (Dw_ib_7) could be isolated at high ionic strength and was identified as a *Pseudoalteromonas* sp.

An eukaryote identified in the water column at low ionic strength was found to have a 97% identity with the dinoflagellate *Woloszynskia cincta* of the *Dinophyceae*. *W. cincta* has only been detected in marine and freshwater systems [27].

#### Sediment isolates

During the dry season cracking of the salt crust was rare; therefore, sediments kept humidity and the oxygen profile did not show signs of ventilation. Different colonies were obtained inoculating sediment samples from different depths using enrichment media for heterotrophic microorganisms. The analysis of the 16S rRNA gene sequence of isolated colonies from the rainy season revealed that *Paenibacillus* sp. from the *Firmicutes* and *Arthrobacter phenanthrenivorans* from the *Actinobacteria* (Table 2) were present in the sediment*.*

The bacterial isolates from the dry season sediments were identified as members of the *Gammaproteobacteria*, with the exception of isolate Ds_ib_4 (*Falsibacillus* sp. in *Firmicutes*). The rest of the isolates were identified as members of the genus *Idiomarina, Pseudoidimarina, Halomonas, Marinobacter*. Despite the fact that sediment samples were inoculated on plates with haloarchaeal media and some colonies showed the presence of characteristic pigments, none of them could be isolated in further purification steps probably due to the media composition (low concentration of sulfate) or probably because the temperature of incubation was far from the optimal.

A fungus was isolated in potato-dextrose-agar (PDA) plates inoculated with a sediment sample from the dry season and was identified as a member of the genus *Aspergillus*.

### Analysis of uncultivated microbes

#### 16S rRNA gene cloning from water samples

**Figure 3:**
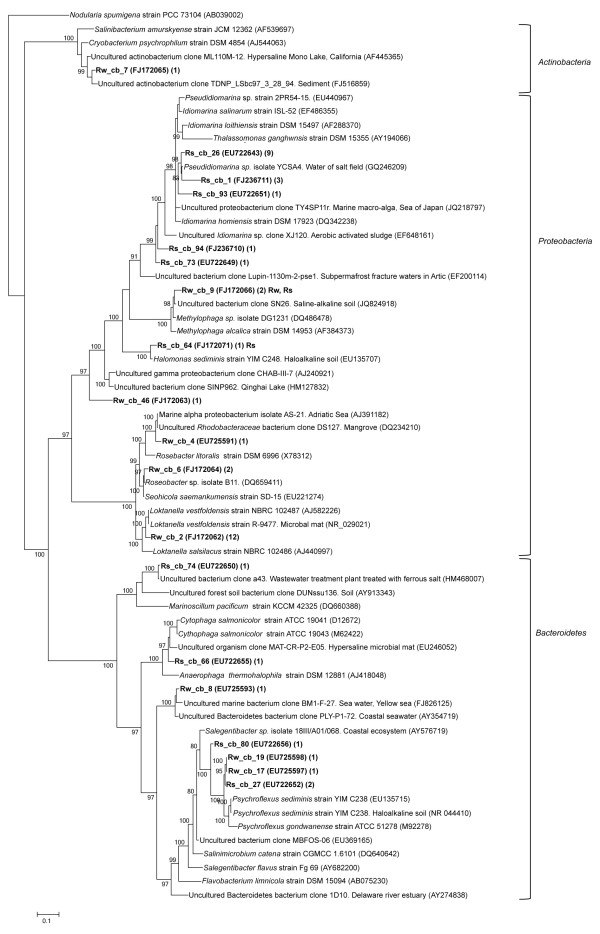
**Phylogenetic tree based on 16S rRNA sequences bacterial clones from Tirez water column and sediment from the rainy season.** The tree topology was constructed with Mr. Bayes. Percentage of probability support in main nodes is indicated if ≥80%. The scale bar represents expected changes per site. Highlighted phylotypes in bold type are prefixed by Rw (rainy water), Rs (rainy sediment), Dw (dry water) and Ds (dry sediment) and followed by cb (clone bacteria) and ID number. The access number and the number of collapsed phylotypes assigned to OTU with ≥97% identity is indicated in parenthesis.

More than half of the clones obtained from the bacterial library of 16S rRNA gene sequences from water column samples of the rainy season had sequences similar to *Alphaproteobacteria* (Figure 3). Most of the clones were affiliated with *Loktanella vestfoldensis* and aggregated in one OTU with 12 phylotypes (a posterior probability (pp) of 100% supported this clade). The remaining phylotypes, Rw_cb_4 and Rw_cb_6, formed a cluster with *Roseobacteraceae* sequences (100% pp) (Figure 3). The phylotype Rw_cb_9 was affiliated with the uncultured bacterium clone SN26 (JQ824918) in the *Methylophaga* clade of the *Gammaproteobacteria* (100% pp). The phylotype Rw_cb_46 did not clustered at species level within *Gammaproteobacteria* by phylogenetic analysis, this view was confirmed with BLASTN and *Classifier* tools since it presented a similarity of 91% with the uncultured bacterium clone SN26 (JQ824918) obtained from an haloalkaline soil (Table 1).

Also, four clones retrieved from the low salinity water column were affiliated with *Bacteroidetes* (genus *Psychroflexus* and uncultured bacterium clones) and *Actinobacteria* phyla (uncultured actinobacterium clone).

**Figure 4:**
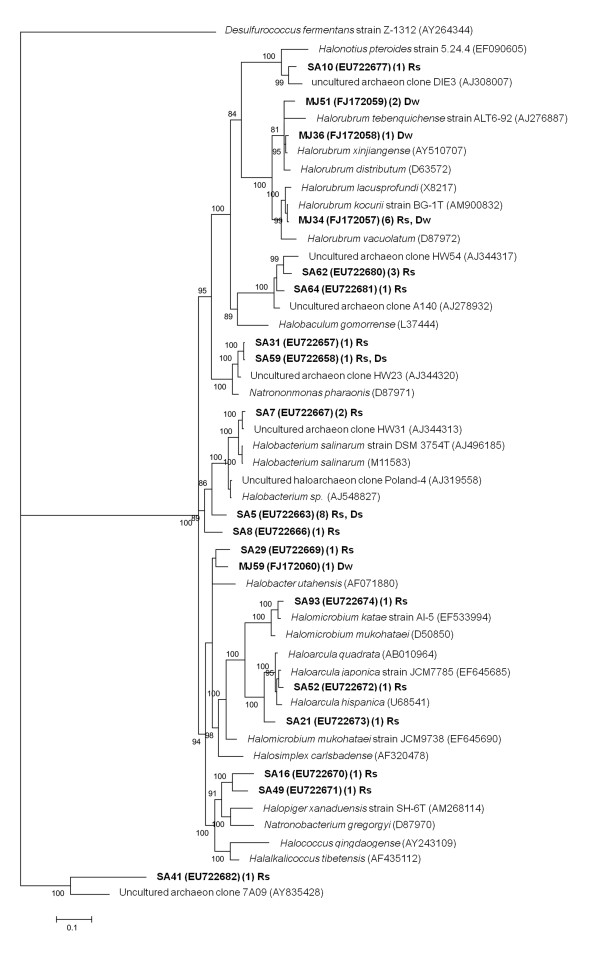
**Phylogenetic tree based on 16S rRNA sequences archaeal clones from Tirez water column and sediments from the rainy and dry seasons.** The tree topology was constructed with Mr. Bayes. Percentage of probability support in main nodes is indicated if ≥80%. The scale bar represents expected changes per site. Highlighted phylotypes in bold type are prefixed by Rw (rainy water), Rs (rainy sediment), Dw (dry water) and Ds (dry sediment) and followed by cb (clone bacteria) and ID number. The number of collapsed phylotypes assigned to OTU with ≥97% identity is indicated in parenthesis.

In the archaeal clone library from the 33% salinity brine a substantial proportion of clone sequences combined three OTUs clustering with the *Halorubrum* genus (Figure 4), with a pp of 100%. One exception was the Dw_ca_59 clone, which was affiliated to an Uncultured euryarchaeote clone SFH1E051 (FN391283) (Figure 4).

Although DNA was successfully extracted from water samples with low salinity, attempts to obtain amplified products with archaeal primers were unsuccessful. This suggests that there were undetectable levels of halophilic archaea in these conditions, probably due to their low tolerance to low salinity concentrations [28].

#### DGGE analysis of water samples

**Figure 5:**
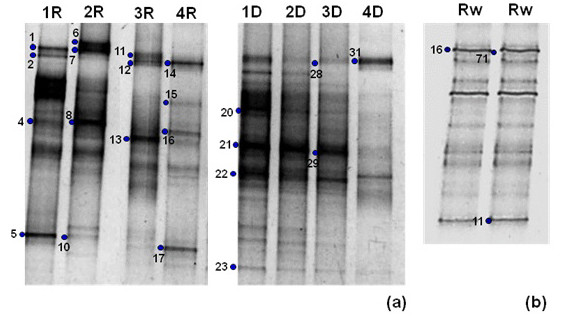
**Denaturing gradient gel electrophoresis (DGGE) band patterns with specific primers for *Bacteria *16S rRNA gene fragment in sediment (a) and water (b) samples.** Band patterns resulted from 30-70% **(a)** and 40-70% **(b)** denaturing gradients. Sediment patterns are D for dry and R for rainy seasons, and their respective depths: 1) surface (0–5 cm), 2) 5–15 cm, 3) 15–25 cm and 4) 25–35 cm. The water DGGE pattern is derived from rainy samples. Bands with numbers were excised from the gels and sequenced. Sequences were identified by the prefix describing the community sampled (Rw, Rs, Dw and Ds), technique used (db for DGGE and bacteria) and ID number.

The genomic DNA from the rainy season water column samples was amplified with the bacterial primer pair 341fGC-907r and resolved by DGGE using a denaturing gradient of 30-70% (Figure 5). The band pattern was reproducible and the most prominent bands were sequenced, showing high similarity with members of *Flavobacteriaceae* and an uncultured Actinomycetales organism (Table 2).

#### Cloning from sediment samples

Samples for cloning from both the dry and the rainy season, were taken at 10 cm because maximum values of biomass (DAPI stain) and metabolic activities (sulfate reduction) were detected at this depth [29]. Fourteen percent of the phylotypes from the rainy season clustered with different *Idiomarina* and *Pseudoidiomarina* species from the *Gammaproteobacteria* (Figure 3, Table 2), representing the most abundant group. The rest of the phylotypes clustered showing higher similarity values with type species sequences of *Halomonas* and *Methylophaga* (*Gammaproteobacteria*). The *Bacteroidetes* phylum was represented by members of the *Anaerophaga*-*Marinilabilia* clade. *Flavobacteria* was represented by members of the *Psychroflexus* and *Microscilla* genera (Figure 3). The bacterial clone library from the dry season sediments yielded members of the *Idiomarina*, *Marinobacter* and *Halomonas* genera within the class *Gammaproteobacteria*.

Archaeal communities in sediments show higher diversity than water samples. About 40% of archaeal clones from both rainy and dry season sediments showed high similarity with *Halobacterium* species. *Halobacterium* species, commonly present in high salinity environments, here were identified by phylogeny with a pp of 100% (Figure 4). The other archaeal phylotypes from the wet season sediments were integrated within *Haloarcula, Natronomonas*, *Halorubrum* and *Natronobacterium-Halopiger* clades. Phylotypes identified as members of the *Natronobacterium* genus were also detected in sediments from the dry season (Table 2). The clone Rs_ca_41 (EU722682) showed some similarity to an uncultured archaeon clone Discovery_a from an hydrothermal brine system in the Read Sea (HQ530525) (Table 2) and by phylogenetic analysis it fell within the Candidate division MSBL1 clade (Figure 4).

#### DGGE analysis of sediment samples

Band patterns were obtained from a denaturing gradient of 40-70% for sediments from rainy and dry season (Figure 5). From a total of 15 identified bacterial bands from the rainy season, five phylotypes belonged to the *Flavobacteriaceae*. Also members of the *Bacillaceae* were detected. Of the *Gammaproteobacteria* the frequently observed members of the genus *Halomonas*, harbouring only halophiles, members of the sulfur oxidizing bacteria *Thioalkalimicrobium* and the heterotroph *Marinobacter* were identified. One band was identified by the tool *Classifier* as a *Desulfohalobiaceae* member a sulfate reducing halophilic group of the *Deltaproteobacteria* class (Table 2).

A total of seven bands were obtained from the dry season sediment sample. Their sequences indicated phylotypes closely related with members of the phylum *Bacteroidetes* (*Muricauda flavescens*, unidentified *Bacteroidetes*) and an unidentified *Sphingobacteria*. Of the *Gammaproteobacteria,* members of the halophilic genus *Halomonas* were identified and the band Rs_db_29 was related with the *Clostridiaceae*.

**Figure 6:**
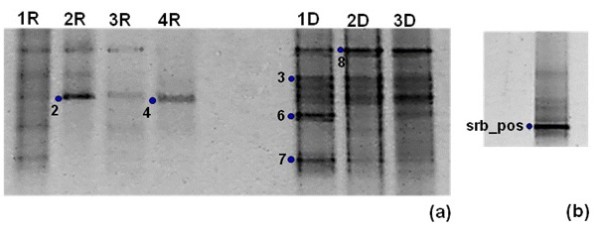
**Denaturing gradient gel electrophoresis (DGGE) band patterns obtained with specific primers for 16S rRNA gene fragments; from *Archaea *in sediment samples (a) and *Deltaproteobacteria *in cultures (b).** The band patterns resulted from 40-70% **(a)** and 30-50% **(b)** denaturing gradients. The patterns are from two different seasons, rainy (R) and dry (D), and their respective depths are: 1) surface (0–5 cm), 2) 5–15 cm, 3) 15–25 cm and 4) 25–35 cm. Bands with numbers were excised from the gels and sequenced. Sequences were identified by the prefix describing the community sampled (Rw, Rs, Dw and Ds), technique used (da for DGGE and archaea) and ID number.

A DGGE pattern was obtained using a 40-70% denaturing gradient of the amplification product of sediment samples using the archaeal primers 344f and 915rGC (Figure 6). The partial 16S rRNA sequence of the main bands allowed us to identify members of the strict halophilic genera *Halobacterium* and *Natronomonas* in samples from both types of sediments, and a species of the genus *Archaeoglobus* in sediments from the wet season (Table 2).

## Discussion

### Physico-chemical conditions

The circumneutral pH of the Tirez lagoon could be the result of the low concentration of carbonates in relation to the concentration of calcium. The difference in pH between the water column and the sediments is probably due to the difference in the ionic concentrations between both phases. In summer, the relative abundance of the most numerous cations of water was as follows: Na+K>Mg>Ca therefore, a low quantity of CO_3_^2-^ is sequestered by carbonate precipitation, which leads to a slightly alkaline pH (Table 1). On the contrary, in the sediments the bivalent cations dominate precipitating the CO_3_^2-^ and resulting in a neutral pH.

The ratio of Cl^-^ to SO_4_^2-^ underlines the athalassohaline characteristic of the Tirez lagoon. The relationship between chloride and sulfate increases from the rainy to the dry season as a consequence of the sequestration of sulfate in different salts (*i.e.* epsomite, mirabilite, thenardite, hexahydrite and bloedite) that precipitate before halite does.

Methanogens make a living in habitats where electron acceptors such as O_2_, NO_3_^-^, Fe^3+^, and SO_4_^2-^ are limiting [30]. Under the sulfated conditions prevailing in Tirez methanogens might be excluded. On the other hand, the negative redox potential values detected in the sediments (Table 1), especially in the rainy season, are not low enough to allow methanogenesis to proceed, being necessary a redox potential lower than -330 mV [31]. Such conditions are enough to justify the lack of evidence of methanogenic archaea by 16S rRNA gene sequencing. However, hydrogenotrophic methanogens have been reported in Tirez lagoon [29].

The negative redox potential values detected in the sediments (Table 1), especially in the rainy season, are not low enough to allow methanogenesis to proceed, being necessary a redox potential lower than -330 mV [31]. This result strongly suggests that measured redox potentials might be a gross estimate of the sediment potentials and that microniches with appropriate physico-chemical conditions must develop in those sediments to facilitate the growth of these strict anaerobic microorganisms.

### Microbial diversity

After comparing the class richness among three domains it was evident that Bacteria was the richest domain given that bacteria classes represented 50% of all classes detected. *Proteobacteria* was the most common *phylum* and accounted for the largest OTU fraction (31%) in all the phases analyzed. Thus, in Tirez, members of the *Proteobacteria* class were the best-represented group of *Bacteria* in the range of salinities studied, diverging from other characterized athalassohaline systems where the dominant role was played by members of the *Bacteroidetes* group [15,32]. Frequently, *Salinibacter ruber* is recognized as a common and abundant bacterium inhabitant of hypersaline environments, including the athalassohaline lake Chaka [32]. However, this microorganism has not been detected in Tirez lagoon in the different seasons and phases analyzed, which suggests a more complex distribution of this species [33].

### Water

Most of the oxygenic photosynthetic organisms were detected and identified by morphotype analysis. The *Chlorophyta* and the *Cyanobacteria* detected are frequently found in hypersaline environments. These microorganisms play an important role in the global carbon and mineral cycles of hypersaline environments [34]. During the dry season, water evaporates promoting massive salt precipitation. However, a thin 3 mm layer of cyanobacteria could be observed between the sediment and the salt crust. Although competition with macrophytes is practically nonexistent at these hypersaline conditions, the cyanobacterial layer becomes thin and ephemeral. One possible explanation proposed by Guerrero and de Wit is that thick cohesive mats are usually associated with permanently covered sediments, in contrast to the thin mats that are temporally submerged, as in our case [35]. Another possible explanation is the Tirez fine sediment granulometry, dominated by particles of ≤0.002 mm, which have been described as the possible cause for the generation of thinner mats [36].

Though a prevalence of *Loktanella vestfoldensis* sequences among clone libraries has not been reported in other hypersaline systems, *Loktanella* sp. has been found in cold saline groundwater springs (Axel Heiberg Island, Canada) rich in sulfate [37]. It is remarkably that the genera *Marinobacter* and *Halomonas* have not been reported in other hypersaline systems rich in sulfate.

The presence of *Woloszynskia cincta* in the wet season seems to be a peculiarity of this environment since there is no report of this species in hypersaline systems. Indeed, there are few reports of dinoflagellates in hypersaline environments [38]. *W. sincta* is absent at high osmolarity conditions, it is possible to explain its survival until the next season by cyst formation [39]. On the other hand, the algae *Dunaliella* sp. is present in dry and flooded seasons. It has been shown that *Dunaliella tertiolecta* growth is inhibited by Mg^2+^ salts when compared with Na^+^ salts [40]. This response is of interest because Mg^2+^ is present in high concentrations in Tirez (Figure 1). However, the proliferation of *Dunaliella* sp. in Tirez lagoon is not entirely surprising since this genus has been reported in the Dead Sea [41] under high concentrations of Mg^2+^. Fuji [40] argues that the ability of *D. tertiolecta* to grow in a MgSO_4_ medium may be related to the high intracellular concentration of SO_4_^2-^. Therefore, the high sulfate concentration characteristic of Tirez might have positive consequences on *Dunaliella* sp. growth despite the high concentrations of bivalent cations.

### Sediments

Most ecological studies on hypersaline ecosystems are focused on the aqueous phase. Therefore, the following data about bacteria and archaea present in sediments is remarkable in terms of diversity of halophiles.

Firstly, 16S rRNA gene sequence techniques showed a predominance of *Haloarchaea*, particularly members of the *Halobacteriaceae*, which can grow well heterotrophically in the dark [42]. Most of the identified bacteria are described as heterotrophs, with *Gammaproteobacteria* being the dominant taxa followed by members of the *Flavobacteria* class.

The thermophilic sulfate reducing *Archaea Archaeoglobus* was detected in Tirez sediments. Interestingly, there are previous evidences of thermophilic genus (*Thermoplasmatales*) described in other hypersaline environments [14,43,44]. To understand this ecological singularity, a contingent adaptation of thermophilic microorganisms to the high osmolarity conditions founded in Tirez should be considered. Thermophilic microorganisms succeeded in stabilizing intracellular macromolecules by the synthesis and/or accumulation of compatible solutes [45,46]. This physiological adaptation is an absolute requirement for organisms under osmotic stress [47]. In fact, the synthesis of compatible solutes has been reported in species of *Archaeoglobus*[48].

Another interesting case was *Desulfohalobium*, a halophilic sulfate reducing bacterium that was identified by PCR-DGGE. Although sulfate reducing bacteria have been detected previously in thalassohaline environments [49], this is the first report of this genus in an athalassohaline sulfate-rich ecosystem.

Microorganisms of the functional groups methanogens, sulfur oxidizers and sulfate reducers have been detected in sediment and by molecular biology techniques and enrichment [29]. Interestingly, methanogenic archaea and sulfur oxidizing bacteria were undetected by PCR-cloning of the gene 16S rRNA. Moreover, the sulfate reducing bacteria here detected by 16S rRNA (*Desulfohalobium* sp.) does not coincide with those encountered with the functional gene marker Apr, i.e. *Desulfonema* and *Desulfonatronovibrio*[29]. This inconsistence between gene markers has been reported previously [50].

## Conclusion

Culture-dependent and –independent techniques were used to examine the microbial diversity of the water column and the sediments in an athalassohaline lagoon, Tirez, from La Mancha (Central Spain). All the phases of the lagoon are inhabited by an abundance of microorganisms, including representatives of the three domains: *Eukarya*, *Bacteria* and *Archaea*.

A difference in community structures was observed between the water column and the sediments. The cyanobacteria occurred mainly in water column. Along with *Haloarchaea*, members of the *Proteobacteria* were well represented in both phases. *Gammaproteobacteria* are the dominant sequences in the sediments. Sulfate reducers were detected in the anoxic part of the sediments. These results lead to the conclusion that extreme concentrations of sulfate might have an effect on the microbial diversity of the habitat that remains to be proved by quantitative analysis.

## Methods

### Sampling

Samples were taken in triplicate on February and July 2005. Water and sediments were obtained from the same area of the lake. Water samples were taken aprox. 10 cm above sediments using sterile 50 mL Falcon tubes and kept at 4°C (for 4 h) until processed. In the dry season 50 mL sterile syringes were used for brine collection. For core extraction a Ring Kit core-sampler (Eijkelkamp Agrisearch equipment, The Netherlands) for soft soil was used. The sampler was inserted down to 40 cm, the core was kept at 4°C until further processing. Samples were used to inoculate cultures in triplicate and for molecular analysis

### Physico-chemical characterization

For water samples, *in situ* temperature, pH, Eh and dissolved oxygen were measured using a multi-parametric probe (YSI 556 MPS, YSI Environmental). Eh and pH along the sediment cores were measured with a probe connected to a potentiometer Orion Model 290A+Thermo Orion (Thermo Fisher Scientific) calibrated at high ionic strength using equivalent Na_2_SO_4_ solutions, and dissolved oxygen and temperature with a Syland TM model Simplair*.* Elemental analysis was performed by TXRF (Extra-II) and ICP-MS (ELAN-6000 PE-Sciex) instruments and ionic chromatography with an IC Dionex DX-600 apparatus. Ion data from Tirez lagoon and other saline systems were used to build a ternary diagram using the software *ProSim Ternary Diagram* (ProSim, France).

### Microscopic examination and cell enumeration

Samples were fixed with formaldehyde at a final concentration of 4% (v/v). The identification of algae, *Cyanobacteria*, *Arthropoda*, *Rotifera* and *Fungi* was carried out by microscopic observation of fixed samples using a Zeiss Axiovert 200M microscope coupled to a CCD camera.

To quantify cell numbers, preparations were stained with 4′, 6′-diamino-2-phenylindole (DAPI), Molecular Probes (Invitrogen), as previously described [51] and counted under a Zeiss Axiovert 200M microscope.

### Microorganism isolation and culture

Sediment samples were dispersed in 1× PBS (0.1 M NaCl, 2 mM KCl, 4 mM Na_2_HPO_4_, pH 7.4) and the suspension used to inoculate different media plates. Each plate was inoculated with 100 μl of sediment slurry. For isolation of *Cyanobacteria*, BG11 medium plates [52] were inoculated and incubated at 20°C for up to 3–4 weeks under a 16:8 light:dark cycle at 150 mmol photons m^2^s^-1^ irradiance and a temperature of 19°C. Fungi were isolated in PDA medium (potato-dextrose-agar) containing 0.050 mg/mL streptomycin and 0.1 mg/mL ampicillin and incubated at 30°C. Heterotrophic microorganisms were isolated on marine agar (Difco, Marine Broth 2216) and media for halophilic strains prepared with salts obtained from water of Tirez lagoon (rainy season) and crystallized with vacuum at room temperature. Salt composition was determined by ionic chromatography with an IC Dionex DX-600 apparatus. Ionic composition was as follows (ppm): Na^+^-K^+^ (8140), Ca^2+^ (1091), Mg^2+^ (4602), NH4^+^ (53.6), Cl^-^ (258) and SO_4_^2-^ (6695). The crystallized salts were dissolved at a final concentration of 10, 20 or 30% (w/v). Dissolved salts were enriched with yeast extract (<0.5 g/L) and glycerol (<0.5 g/L) as reported by Bolhuis *et al*. [53]. Representative individual colonies from each medium were reinoculated in the same growth condition. All plate isolates were transferred to liquid media to obtain enough biomass to allow DNA extraction for molecular analysis. Sulfate-reducing bacteria were grown in anaerobic SRB medium modified from Raskin *et al*. [54] and supplemented with 500 mg/L L-cysteine, as reductive agent, and the following organic substrates: 250 mg/L yeast extract, 770 mg/L glutamic acid, 15 mg/L glycine, 250 mg/L peptone, 14 mM methanol and 27 mM methylamine.

### Molecular methods

#### DNA extraction

To collect cells 100 mL of water samples were filtered onto 0.22 μm polycarbonate filters (Millipore). Sediment samples were sonicated in 1× PBS during 3 min at 4°C and power of 73 w/cycle (Labsonic B. Braun, Germany), before DNA extraction. In all cases DNA was extracted using Power Soil DNA Isolation Kit (MoBio, Labs. Inc., Solana Beach, CA), following manufacturer’s directions and purified using a DNA purification *JetQuick* kit (Genomed).

For extraction of dinoflagellate DNA, microalgal cells were picked, one by one from the water samples with a microcapillary pipette under an inverted microscope (Zeiss A at 60× and 400× magnification), washed 2–3 times using sterile 1× PBS, placed (with as little liquid as possible) in 0.2 mL Eppendorf tubes containing 5 μl of lysis buffer (0.005% SDS and 400 ng/μL Proteinase K) and treated as in the procedure described by Kai *et al.*[55].

### PCR conditions for rRNA gene amplification

Amplifications were performed using a Thermal Cycler 2720 (Applied Biosystems) in a final volume of 50 μL, each containing: 1 mM of dNTP, 3 mM MgCl_2_, 1 mM of each primer, 1× PCR buffer and 0.025 u/μL Taq DNA Polimerase (AmpliTaq DNA Polymerase, Roche Molecular Systems). DNA was added in a volume of 3 μL, containing about 1–5 ng of template. Bacterial 16S rDNA was amplified using primers 27f [56] and 1492mr (5′-TACGGYTACCTTGTTACGACTT-3′) modified from [57] (annealing 57°C; 30 cycles). The 25f [58] and 1492mr primers were used for *Archaea* domain (52°C; 27 cycles). Both 16S rDNA amplification procedures consisted of initial denaturation (94°C for 10 min) followed by the above-indicated number of cycles of denaturation (94°C for 1 min), annealing (at the temperatures indicated above for 1 min) and extension (72°C for 3 min) followed by a final cycle of extension (72°C for 10 min). PCR amplification of 18S rDNAs were performed using the primer pair Euk1Af-Euka516 as described in [59].

For dinoflagellates an 18S rDNA fragment was amplified using dinoflagellate-specific primers Dino18SF1m (5′-AAGGGTTGTGTTTATTAGNTACAGAAC-3′) modified from [60] and 18ScomR1 [61]. The reaction was performed with an initial denaturation (94°C for 5 min), followed by 30 cycles of denaturation at 94°C for 1 min, annealing at 56°C for 1 min, and extension at 72°C for 3 min.

PCR amplifications for DGGE analysis of rRNA gene fragments were performed for *Bacteria* using the primers 341fGC [62] and 907r [56] while for *Archaea* the region between the primers 344fGC [62] and 915r [63] was used. Amplification conditions for *Bacteria* were the following: 94°C for 7 min, 35 cycles of 94°C for 45 s, 49°C for 45 s and 72°C for 1.5 min, and a final extension of 72°C for 10 min. For *Archaea* primers: 94°C for 5 min, 32 cycles of 94°C for 30 s, 54°C for 1 min and 72°C for 1 min, and a final extension of 72°C for 10 min. A 16S rRNA *Deltaproteobacteria*-specific region was amplified as described in [50] using the 385fGC-907r primer pair. Functional gene primers used for detection of sulfate reducing and methanogenic activities are described in [29]. In PCR reactions GC was equivalent to a 40 bp GC clamp at the 5′ end to prevent complete melting of the DNA fragments.

### Cloning of 16S rRNA

PCR amplified products were cloned using the TOPO TA Cloning kit (Invitrogen Corporation, California) according to the manufacturer’s indications. From each clone library putative positive transformants were randomly sampled to perform minipreps according to standard alkaline lysis protocols.

### Denaturing gradient gel electrophoresis

DGGE analysis of PCR-amplified 16S rRNA gene fragments using a 30-70%, 40-70%, 40-70% and 30-50% gradients was performed as described by Muyzer *et al*. [64] using a D-Code Universal Detection System (BioRad Laboratories). PCR samples were loaded onto 8% (w/v) polyacrylamide gels in 1× TAE buffer (20 mM Tris, 10 mM acetate, 0.5 mM Na-EDTA, pH 7.4). Electrophoresis was carried out at 60°C, at a constant voltage of 200 V for 4.5 h. After electrophoresis, the gel was stained for 15 min with ethidium bromide (0.5 μg/mL), rinsed in distilled water for 30 min and photographed with a Polaroid Kodak digital 16 camera. DGGE bands were excised from the gel under UV light and eluted in 50 μl of milliQ water overnight at 4°C. An aliquot of 3 μL was taken from each eluted sample and re-amplified by PCR in the conditions described above. The primers used for re-amplifications were the corresponding ones used in the first amplification but without the tailing sequence.

### Sequence analysis

PCR products from DGGE gel bands and plasmid DNAs containing inserts were sequenced with the primers used for amplification and the pair M13F/M13R, respectively using an ABI PRISM Big Dye Terminator Cycle Sequencing Ready Reaction Kit (ABI) and an Applied Biosystem ABI 310 (PE Applied Biosystems, Foster City, California, USA) automated sequencer. Chromatograms were transformed into contiguous sequences combining *FinchTV* (http://www.geospiza.com/finchtv) and *GeneDoc* (http://www.nrbsc.org) tools. Chimeric sequences were identified by using *Mallard*[65]. The 16S and 18S rRNA sequences obtained from DGGE bands, culture isolates and clones were collapsed into OTUs by similarity analysis using *FastGroup II* (http://fastgroup.sdsu.edu). OTUs were compared with those available in GenBank (NCBI) and Ribosomal Database Project (RDBP) to identify them using the Basic Local Aligment Search Tool Nucleotide (BLASTN) and *Classifier* algorithms, respectively. Similarity analysis was performed with *FastGroup II* (http://fastgroup.sdsu.edu)

### Phylogenetic analysis

The 16S rRNA gene sequences with a length ≥ 1300 bp were aligned with representative ones published in Bergey’s Manual [66] using *ClustalX*[67] using default parameters. Alignments were optimized manually using *BioEdit* version 7.0.5.3 [68]. A similarity matrix was calculated by using the similarity matrix tool located at the Ribosomal Database Project homepage (http://rdp.cme.msu.edu/cgis/phylip.cgi). Operational Taxonomic Units (OTUs) were defined as sequences obtained from the same technique that showed a similarity more than 97% with each other. Similarity analysis was performed with *FastGroup II* (http://fastgroup.sdsu.edu). Alignments of OTUs obtained from cloning were exported to test different nucleotide substitution models using *Phylip* available in http://phylemon.bioinfo.cipf.es. GTR was consequently the optimal model. Posterior probability and topology of the phylogenetic trees were obtained with *Mr. Bayes* version 3.1.2 [69] defining the parameters GTR+I+G. Tree analysis was a consensus of 5x10^5^ generations (SD=0.02) in *Archaea* and 2.5×10^5^ generations in *Bacteria* (SD=0.04), in both cases it was performed a “burnin” of 50%.

### GenBank sequence accession numbers

The SSU rRNA fragment gene sequences were deposited in the GenBank database under accession numbers, EU734574, EU725589-EU725602, EU722643-EU722714, FJ172052-FJ172100 and FJ236710-FJ236714. Prefixes of sequences describe: community sampled (Rw for rainy water, Rs for rainy sediment, Dw for dry water and Ds for dry sediment), technique used (c for clone, i for isolate and d for DGGE-band) and domain (b for *Bacteria* and a for *Archaea*) and identification number.

## Competing interests

Authors declare that they have no competing interests.

## Authors’ contributions

LM, IM and RA conceived and coordinated the study, participated in the design, field sampling and analysis of the results and drafted the manuscript. CV and NR participated in the design of the study, field sampling, the geochemical characterization of the samples, and the isolation and identification of the microorganisms. All authors revised critically the draft of the paper and approved the final manuscript.

## Supplementary Material

Additional file 1: Figure S1 Microorganisms identified by morphology in Tirez lagoon. (a) *Anabaena* sp., (b) *Microcoleus chthonoplastes*, (c) Diatoms, (d) *Aspergillus* sp. (e) *Nodularia* sp., and (f) *Leptolyngbya* sp. Click here for file

## References

[B1] Sorokin DY Kuenen JG Muyzer G The microbial sulfur cycle at extremely haloalkaline conditions of soda lakes Front Microbiol 2011 2 44 http://www.ncbi.nlm.nih.gov/pmc/articles/PMC3128939/21747784 10.3389/fmicb.2011.00044 PMC3128939

[B2] vd Wielen PWJJ Bolhuis H Borin S Daffonchio D Corselli C Giuliano L D’Auria G de Lange GJ Huebner A Varnavas SP Thomson J Tamburini C Marty D McGenity TJ Timmis KN BioDeep Scientific Party The Enigma of Prokaryotic Life in Deep Hypersaline Anoxic Basins Science 2005 307 121 123 http://www.sciencemag.org/cgi/content/abstract/307/5706/121 10.1126/science.1103569 15637281

[B3] Porter D Roychoudhury AN Cowan D Dissimilatory sulfate reduction in hypersaline coastal pans: Activity across a salinity gradient Geochim Cosmochim Acta 2007 71 5102 5116 http://www.sciencedirect.com/science/article/pii/S0016703707004954 10.1016/j.gca.2007.08.023

[B4] Borin S Brusetti L Mapelli F D’Auria G Brusa T Marzorati M Rizzi A Yakimov M Marty D De Lange GJ Sulfur cycling and methanogenesis primarily drive microbial colonization of the highly sulfidic Urania deep hypersaline basin Proc Natl Acad Sci U S A 2009 106 9151 9156 http://www.pnas.org/content/106/23/9151.short 10.1073/pnas.0811984106 19470485 PMC2685740

[B5] Sorokin DY Zacharova EE Pimenov NV Tourova TP Panteleeva AN Muyzer G Sulfidogenesis in hypersaline chloride-sulfate lakes of Kulunda Steppe (Altai, Russia) FEMS Microbiol Ecol 2012 79 445 453 http://www.ncbi.nlm.nih.gov/pubmed/22092787 10.1111/j.1574-6941.2011.01228.x 22092787

[B6] Foti M Sorokin DY Lomans B Mussman M Zacharova EE Pimenov NV Kuenen JG Muyzer G Diversity, Activity, and Abundance of Sulfate-Reducing Bacteria in Saline and Hypersaline Soda Lakes Appl Environ Microbiol 2007 73 2093 2100 http://www.ncbi.nlm.nih.gov/pmc/articles/PMC1855663/10.1128/AEM.02622-06 17308191 PMC1855663

[B7] Mesbah NM Abou-El-Ela SH Wiegel J Novel and unexpected prokaryotic diversity in water and sediments of the alkaline, hypersaline lakes of the Wadi An Natrun, Egypt Microb Ecol 2007 54 598 617 http://www.ncbi.nlm.nih.gov/pubmed/17450395 10.1007/s00248-006-9193-y 17450395

[B8] Eugster HP Hardie LA Lerman A Saline lake Physics and chemistry of lakes 1978 Springer-Verlag 237 293

[B9] Youssef NH Ashlock-Savage KN Elshahed MS Phylogenetic Diversities and Community Structure of Members of the Extremely Halophilic Archaea (Order Halobacteriales) in Multiple Saline Sediment Habitats Appl Environ Microbiol 2012 78 1332 1344 http://aem.asm.org/content/78/5/1332.long 10.1128/AEM.07420-11 22179255 PMC3294467

[B10] Kushner DJ Woese CR, Wolfe RS The *Halobacteriaceae* The Bacteria 1985 3 London: Academic Press 171 214

[B11] Maturrano L Santos F Rossello-Mora R Anton J Microbial Diversity in Maras Salterns, a Hypersaline Environment in the Peruvian Andes Appl Environ Microbiol 2006 72 3887 3895 http://aem.asm.org/cgi/content/abstract/72/6/3887 10.1128/AEM.02214-05 16751493 PMC1489619

[B12] Dong H Zhang G Jiang H Yu B Chapman LR Lucas CR Fields MW Microbial Diversity in Sediments of Saline Qinghai Lake, China: Linking Geochemical Controls to Microbial Ecology Microb Ecol 2006 51 65 82 http://www.springerlink.com/content/y624u261904148mw/10.1007/s00248-005-0228-6 16400537

[B13] Litchfield CD **Saline Lakes** Encyclopedia of Geobiology 2011 Springer: Reitner J, Thiel V. Dordrecht 765 769

[B14] Jiang H Dong H Yu B Liu X Li Y Ji S Zhang CL Microbial response to salinity change in Lake Chaka, a hypersaline lake on Tibetan plateau Environ Microbiol 2007 9 2603 2621 http://www3.interscience.wiley.com/journal/118491062/abstract 10.1111/j.1462-2920.2007.01377.x 17803783

[B15] Demergasso C Casamayor EO Chong G Galleguillos P Escudero L Pedrós-Alió C Distribution of prokaryotic genetic diversity in athalassohaline lakes of Atacama Desert, Northern Chile FEMS Microbiol Ecol 2004 48 57 69 http://www.blackwell-synergy.com/doi/abs/10.1016/j.femsec.2003.12.013 10.1016/j.femsec.2003.12.013 19712431

[B16] Escalante AE Eguiarte LE Espinosa-Asuar L Forney LJ Noguez AM Souza Saldivar V Diversity of aquatic prokaryotic communities in the Cuatro Cienegas basin FEMS Microbiol Ecol 2008 65 50 60 http://www.ncbi.nlm.nih.gov/pubmed/18479448 10.1111/j.1574-6941.2008.00496.x 18479448

[B17] Wallmann K Aghib FS Castradori D Cita MB Suess EJ Greinert J Rickert D Sedimentation and formation of secondary menerals in the hypersaline Discovery Basin, eastern Mediterranean Mar Geol 2002 186 9 28 doi:10.1016/S0025-3227(02)00170-6 10.1016/S0025-3227(02)00170-6

[B18] La Cono V Smedile F Bortoluzzi G Arcadi E Maimone G Messina E Borghini M Oliveri E Mazzola S L’Haridon S Unveiling microbial life in new deep-sea hypersaline Lake Thetis. Part I: Prokaryotes and environmental settings Environ Microbiol 2011 13 2250 2268 http://onlinelibrary.wiley.com/doi/10.1111/j.1462-2920.2011.02478.x/abstract 10.1111/j.1462-2920.2011.02478.x 21518212

[B19] Navarro JB Moser DP Flores A Ross C Rosen MR Dong H Zhang G Hedlund BP Bacterial succession within an ephemeral hypereutrophic Mojave Desert playa Lake Microb Ecol 2009 57 307 320 http://www.springerlink.com/content/l54u2843g41580h0/10.1007/s00248-008-9426-3 18758846

[B20] Prieto-Ballesteros O Rodríguez N Kargel JS González-Kessler C Amils R Fernández-Remolar D Tirez Lake as a Terrestrial Analog of Europa Astrobiology 2003 3 863 877 http://www.liebertonline.com/doi/abs/10.1089/153110703322736141 10.1089/153110703322736141 14987487

[B21] de la Peña JA García-Ruiz JM Prieto M Growth features of magnesium and sodium salts in a recent playa lake of La Mancha (Spain) Estudios geol 1982 38 245 257

[B22] de la Peña JA Marfil R La sedimentación salina actual en las lagunas de La Mancha: una síntesis Cuadernos de Geología Ibérica 1986 10 235 270 http://dialnet.unirioja.es/servlet/articulo?codigo=264994

[B23] DasSarma S DasSarma P Halophiles Encyclopedia of Life Sciences 2012 Chichester: John Wiley & Sons Ltd

[B24] Walsh EJ Schröder T Wallace RL Ríos-Arana JV Rico-Martínez R Rotifers from selected inland saline waters in the Chihuahuan Desert of México Saline Systems 2008 2008 4 http://www.salinesystems.org/content/4/1/7/abstract 10.1186/1746-1448-4-7 PMC2441627 18533042

[B25] Ye J McGinnis S Madden TL BLAST: improvements for better sequence analysis Nucleic Acids Res 2006 34 W6 W9 http://www.ncbi.nlm.nih.gov/pmc/articles/PMC1538791/10.1093/nar/gkl164 16845079 PMC1538791

[B26] Cole JR Wang Q Cardenas E Fish J Chai B Farris RJ Kulam-Syed-Mohideen AS McGarrell DM Marsh T Garrity GM Tiedje JM The Ribosomal Database Project: improved alignments and new tools for rRNA analysis Nucleic Acids Res 2009 37 D141 D145 http://www.ncbi.nlm.nih.gov/pmc/articles/PMC2686447/10.1093/nar/gkn879 19004872 PMC2686447

[B27] Kang NS Jeong HJ Yoo YD Yoon EY Lee KH Lee K Kim G Mixotrophy in the newly described phototrophic dinoflagellate Woloszynskia cincta from western Korean waters: feeding mechanism, prey species and effect of prey concentration J Eukaryot Microbiol 2011 58 152 170 http://www.ncbi.nlm.nih.gov/pubmed/21332876 10.1111/j.1550-7408.2011.00531.x 21332876

[B28] Wright AD Phylogenetic relationships within the order Halobacteriales inferred from 16S rRNA gene sequences Int J Syst Evol Microbiol 2006 56 1223 1227 http://ijs.sgmjournals.org/cgi/content/abstract/56/6/1223 10.1099/ijs.0.63776-0 16738095

[B29] Montoya L Lozada-Chavez I Amils R Rodriguez N Marin I The sulfate-rich and extreme saline sediment of the ephemeral tirez lagoon: a biotope for acetoclastic sulfate-reducing bacteria and hydrogenotrophic methanogenic archaea Int J Microbiol 2011 753 758 http://www.hindawi.com/journals/ijmb/2011/753758/10.1155/2011/753758 PMC3170894 21915180

[B30] Liu Y Whitman WB Metabolic, phylogenetic, and ecological diversity of the methanogenic archaea Ann N Y Acad Sci 2008 1125 171 189 http://www.ncbi.nlm.nih.gov/pubmed/18378594 10.1196/annals.1419.019 18378594

[B31] Lange M Ahring BK A comprehensive study into the molecular methodology and molecular biology of methanogenic Archaea FEMS Microbiol Rev 2001 25 553 571 http://www.ncbi.nlm.nih.gov/pubmed/11742691 10.1111/j.1574-6976.2001.tb00591.x 11742691

[B32] Jiang H Dong H Zhang G Yu B Chapman LR Fields MW Microbial Diversity in Water and Sediment of Lake Chaka Appl Environ Microbiol 2006 72 3832 3845 http://aem.asm.org/cgi/content/abstract/72/6/3832 10.1128/AEM.02869-05 16751487 PMC1489620

[B33] Antón J Peña A Santos F Martínez-García M Schmitt-Kopplin P Rosselló-Mora R Distribution, abundance and diversity of the extremely halophilic bacterium *Salinibacter ruber* Saline Systems 2008 4 15 http://www.salinesystems.org/content/4/1/15 10.1186/1746-1448-4-15 18957079 PMC2596770

[B34] Oren A Formation and breakdown of glycine betaine and trimethylamine in hypersaline environments Antonie v Leeuwenhoek 1990 58 291 298 http://www.springerlink.com/content/t832t364771784u0 10.1007/BF00399342 2082817

[B35] Guerrero MC de Wit R Microbial mats in the inland saline lakes of Spain Limnetica 1992 8 197 204 http://www.limnetica.com/Limnetica/Limne08/L08u197_Microbial_mats_in_saline_lakes.pdf

[B36] Pueyo-Mur JJ De la Peña JA Pueyo-Mur JJ Los lagos salinos españoles. Sedimentología, hidroquímica y diagénesis Génesis de Formaciones evaporíticas. Modelos andinos e ibéricos 1991 Barcelona: Universidad de Barcelona 163 192

[B37] Niederberger TD Perreault NN Tille S Lollar BS Lacrampe-Couloume G Andersen D Greer CW Pollard W Whyte LG Microbial characterization of a subzero, hypersaline methane seep in the Canadian High Arctic ISME J 2010 4 1326 1339 http://www.nature.com/ismej/journal/v4/n10/full/ismej201057a.html 10.1038/ismej.2010.57 20445635

[B38] Edgcomb V Orsi W Leslin C Epstein SS Bunge J Jeon S Yakimov MM Behnke A Stoeck T Protistan community patterns within the brine and halocline of deep hypersaline anoxic basins in the eastern Mediterranean Sea Extremophiles 2009 13 151 167 http://www.ncbi.nlm.nih.gov/pubmed/19057844 10.1007/s00792-008-0206-2 19057844

[B39] Owen KC Norris DR Cysts and Life Cycle Considerations of the Thecate Dinoflagellate *Fragilidium* J Coast Res 1985 1 263 266 http://www.jstor.org/stable/4297064

[B40] Fuji S The Growth and Intracellular Ionic Composition of *Dunaliella tertiolecta* in Magnesium-Rich Media Plant and Cell Physiol 1991 32 549 554 http://pcp.oxfordjournals.org/cgi/content/abstract/32/4/549

[B41] Oren A Gunde-Cimerman N, Oren A, Plemenitaš A A century of *Dunaliella* research: 1905–2005 Adaptation of Life at High Salt Concentrations in Archaea, Bacteria, and Eukarya 2005 9 Dordrecht, Netherlands: Springer 491 502 [*Cellular Origin, Life in Extreme Habitats and Astrobiology*]. http://www.ncbi.nlm.nih.gov/pmc/articles/PMC1224875/10.1007/1-4020-3633-7_31

[B42] Oren A Bioenergetic Aspects of Halophilism Microbiol Molec Biol Rev 1999 63 334 348 http://mmbr.highwire.org/cgi/content/abstract/63/2/334 10357854 10.1128/mmbr.63.2.334-348.1999 PMC98969

[B43] Benlloch S López-López A Casamayor EO Øvreås L Goddard V Daae FD Smerdon G Massana R Joint I Thingstad F Pedrós-Alió C Rodríguez-Valera F Prokaryotic genetic diversity throughout the salinity gradient of a coastal solar saltern Environ Microbiol 2002 4 349 360 http://www.blackwell-synergy.com/doi/abs/10.1046/j.1462-2920.2002.00306.x 10.1046/j.1462-2920.2002.00306.x 12071980

[B44] Cytryn E Minz D Oremland RS Cohen Y Distribution and diversity of archaea corresponding to the limnological cycle of a hypersaline stratified lake (Solar Lake, Sinai, Egypt) Appl Environ Microbiol 2000 66 3269 3276 http://aem.asm.org/cgi/content/abstract/66/8/3269 10.1128/AEM.66.8.3269-3276.2000 10919780 PMC92144

[B45] Lamosa P Martins LO da Costa MS Santos H Effects of Temperature, Salinity, and Medium Composition on Compatible Solute Accumulation by *Thermococcus* spp Appl Environ Microbiol 1998 64 3591 3598 http://aem.asm.org/cgi/content/abstract/64/10/3591 9758772 10.1128/aem.64.10.3591-3598.1998 PMC106469

[B46] Roberts MF Organic compatible solutes of halotolerant and halophilic microorganisms Saline Systems 2005 1 1 30 http://www.salinesystems.org/content/1/1/5 10.1186/1746-1448-1-1 16176595 PMC1224877

[B47] Oren A Microbial life at high salt concentrations: phylogenetic and metabolic diversity Saline Systems 2008 4 2 http://www.salinesystems.org/content/4/1/2 10.1186/1746-1448-4-2 18412960 PMC2329653

[B48] Gonçalves LG Huber R da Costa MS Santos H A variant of the hyperthermophile *Archaeoglobus fulgidus* adapted to grow at high salinity FEMS Microbiol Lett 2003 218 239 244 http://www3.interscience.wiley.com/journal/118841256/abstract 10.1111/j.1574-6968.2003.tb11523.x 12586398

[B49] Kjeldsen KU Loy A Jakobsen TF Thomsen TR Wagner M Ingvorsen K Diversity of sulfate-reducing bacteria from an extreme hypersaline sediment, Great Salt Lake (Utah) FEMS Microbiol Ecol 2007 60 287 298 http://www.ncbi.nlm.nih.gov/pubmed/17367515 10.1111/j.1574-6941.2007.00288.x 17367515

[B50] Joulian C Ramsing NB Ingvorsen K Congruent Phylogenies of Most Common Small-Subunit rRNA and Dissimilatory Sulfite Reductase Gene Sequences Retrieved from Estuarine Sediments Appl Environ Microbiol 2001 67 3314 3318 http://aem.asm.org/cgi/content/abstract/67/7/3314 10.1128/AEM.67.7.3314-3318.2001 11425760 PMC93019

[B51] Kepner RL Pratt JR Use of fluorochromes for direct enumeration of total bacteria in environmental samples: past and present Microbiol Reviews 1994 58 603 615 http://mmbr.asm.org/cgi/content/abstract/58/4/603 10.1128/mr.58.4.603-615.1994 PMC372983 7854248

[B52] Rippka R Deruelles J Waterbury J Herdman M Stanier R Generic assignments, strain histories and properties of pure cultures of cyanobacteria J Gen Microbiol 1979 111 1 61 10.1099/00221287-111-1-1

[B53] Bolhuis H te Poele EM Rodríguez-Valera F Isolation and Cultivation of Walsby’s square archaeon Environ Microbiol 2004 6 1287 1291 http://www3.interscience.wiley.com/journal/118811343/abstract 10.1111/j.1462-2920.2004.00692.x 15560825

[B54] Raskin L Rittmann BE Stahl DA Competition and Coexistence of Sulfate-Reducing and Methanogenic Populations in Anaerobic Biofilms Appl Environ Microbiol 1996 62 3847 3857 http://aem.asm.org/cgi/content/abstract/62/10/3847 16535428 10.1128/aem.62.10.3847-3857.1996 PMC1388966

[B55] Kai KL Cheung YK Yeung PKK Wong JTY Development of single-cell PCR methods for the *Raphidophyceae* Harmful Algae 2006 5 649 657 http://www.sciencedirect.com/science/journal/15689883 10.1016/j.hal.2006.01.002

[B56] Brosius J Dull TJ Sleeter DD Noller HF Gene organization and primary structure of a ribosomal RNA operon from *Escherichia coli* J Mol Biol 1981 15 107 127 http://www.sciencedirect.com/science/journal/00222836 7028991 10.1016/0022-2836(81)90508-8

[B57] Lane DJ Stackerbrandt E, Goodfellow M 16S/23S rRNA sequencing Nucleic Acid Techniques in Bacterial Systematics 1991 New York: John Wiley and Sons Ltd 115 175

[B58] DeLong EF Archaea in coastal marine environments Proc Natl Acad Sci U S A 1992 89 5685 5689 http://www.pnas.org/content/89/12/5685.abstract 10.1073/pnas.89.12.5685 1608980 PMC49357

[B59] Diez B Pedrós-Alio C Marsh TL Massana R Application of Denaturing Gradient Gel Electrophoresis (DGGE) To Study the Diversity of Marine Picoeukaryotic Assemblages and Comparison of DGGE with Other Molecular Techniques Appl Environ Microbiol 2001 67 2942 2951 http://aem.asm.org/cgi/content/abstract/67/7/2932 10.1128/AEM.67.7.2942-2951.2001 11425706 PMC92965

[B60] Lin S Zhang HY Miranda L Bhattacharya D Development of a Dinoflagellate-Oriented PCR Primer Set Leads to Detection of Picoplanktonic Dinoflagellates from Long Island Sound Senjie Appl Environ Microbiol 2006 72 5626 5630 http://aem.asm.org/cgi/content/abstract/72/8/5626 10.1128/AEM.00586-06 16885319 PMC1538694

[B61] Zhang H Lin S Phylogeny of dinoflagellates based on mitochondrial cytochrome b and nuclear small subunit rDNA sequence comparisons J Phycol 2005 41 411 420 http://www3.interscience.wiley.com/journal/118646218/abstract 10.1111/j.1529-8817.2005.04168.x

[B62] Baker GC Smith JJ Cowan DA Review and re-analysis of domain-specific 16S primers J Microbiol Methods 2003 55 541 555 http://www.sciencedirect.com/science/journal/01677012 10.1016/j.mimet.2003.08.009 14607398

[B63] Raskin L Stromley JM Rittmann BE Stahl DA Group-specific 16S rRNA hybridization probes to describe natural communities of methanogens Appl Environ Microbiol 1994 60 1232 1240 http://aem.asm.org/cgi/content/abstract/60/4/1232 7517128 10.1128/aem.60.4.1232-1240.1994 PMC201464

[B64] Muyzer G de Waal EC Uitterlinden AG Profiling of complex microbial populations by denaturing gradient gel electrophoresis analysis of polymerase chain reaction-amplified genes coding for 16S rRNA Appl Environ Microbiol 1993 59 695 700 http://aem.asm.org/cgi/content/abstract/59/3/695 7683183 10.1128/aem.59.3.695-700.1993 PMC202176

[B65] Ashelford KE Chuzhanova NA Fry JC Jones AJ Weightman AJ New screening software shows that most recent large 16S rRNA gene clone libraries contain chimeras Appl Environ Microbiol 2006 72 5734 5741 http://aem.asm.org/cgi/content/abstract/72/9/5734 10.1128/AEM.00556-06 16957188 PMC1563593

[B66] Garrity GM Bell JA Lilburn TG Taxonomic Outline of the Prokaryotes. Bergey’s Manual of Systematic Bacteriology 2004 2 Release 5.0; New York: Springer

[B67] Thompson JD Higgins DG Gibson TJ CLUSTAL W: improving the sensitivity of progressive multiple sequence alignment through sequence weighting, position-specific gap penalties and weight matrix choice Nucleic Acids Res 1994 22 4673 4680 http://nar.oxfordjournals.org/cgi/content/abstract/22/22/4673 10.1093/nar/22.22.4673 7984417 PMC308517

[B68] Hall TA BioEdit: a user-friendly biological sequence alignment editor and anlysis program for Windows 95/98/NT Nucl Acids Symp Ser 1999 41 95 98 http://www.mbio.ncsu.edu/JWB/papers/1999Hall1.pdf

[B69] Ronquist F Huelsenbeck JP MrBayes 3: Bayesian phylogenetic inference under mixed models Bioinformatics 2003 19 1572 1574 http://bioinformatics.oxfordjournals.org/cgi/content/abstract/19/12/1572 10.1093/bioinformatics/btg180 12912839

