# Supplementary figures and images for: Microbial community composition of Tirez lagoon (Spain), a highly sulfated athalassohaline environment
Source: Aquat Biosyst. 2013 Oct 2;9:19. doi: 10.1186/2046-9063-9-19 (PMC3852488; doi:10.1186/2046-9063-9-19)

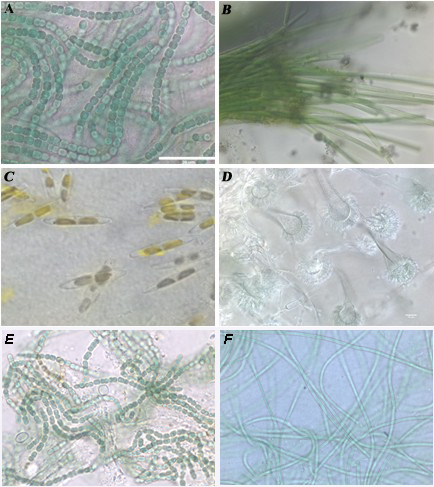

Supplement: Additional file 1: Figure S1 — Microorganisms identified by morphology in Tirez lagoon. (a) Anabaena sp., (b) Microcoleus chthonoplastes, (c) Diatoms, (d) Aspergillus sp. (e) Nodularia sp., and (f) Leptolyngbya sp. [file 2046-9063-9-19-S1.jpeg]
